# Metallofullerenol Sc_3_N@C_80_(OH)_18_: A New Generation Radioprotector Protecting Human Erythrocytes Against Multiple Biochemical Damage Modes Upon Gamma Irradiation, Identifying It as a Scavenger of Short‐ and Long‐Lived Radicals

**DOI:** 10.1002/adhm.202502621

**Published:** 2025-11-13

**Authors:** Jacek Grebowski, Maciej Studzian, Szymon Lekki‐Porebski, Anna Konarska, Marian Wolszczak, Grzegorz Litwinienko, Lukasz Pulaski

**Affiliations:** ^1^ Faculty of Biology and Environmental Protection University of Lodz Pomorska 141/143 Lodz 90‐236 Poland; ^2^ Military Institute of Medicine ‐ National Research Institute Szaserow 128 Warsaw 04‐141 Poland; ^3^ Laboratory of Transcriptional Regulation Institute of Medical Biology PAS Lodowa 106 Lodz 93‐232 Poland; ^4^ BioMedChem Doctoral School of the University of Lodz and the Lodz Institutes of the Polish Academy of Sciences Matejki 21/13 Lodz 93‐237 Poland; ^5^ Institute of Applied Radiation Chemistry Technical University of Lodz Wroblewskiego 15 Lodz 93‐590 Poland; ^6^ Faculty of Chemistry University of Warsaw Pasteura 1 Warsaw 02‐093 Poland

**Keywords:** erythrocytes, metallofullerenol, oxidative damage, radioprotection, radiotherapy

## Abstract

Metallofullerenols (MFs), functionalized endohedral fullerenes, exhibit unique activity by integrating atomic, molecular, and supramolecular levels of matter organization. The antioxidant properties of MFs constitute a novel technology, utilizing these nanocompounds for radioprotection. This integration of nanotechnology with radiological protection may contribute to revolutionary solutions in nuclear safety. Preclinical studies demonstrate low toxicity of MFs and high therapeutic value as redox mediators. In this study, the interaction of the metallofullerenol Sc_3_N@C_80_(OH)_18_ with high‐energy radiation and reactive oxygen species (ROS) is explored, laying the foundation for applications in modern cancer therapy. Radioprotective assessments are conducted on human erythrocytes exposed to gamma (γ) radiation. The rate constant determined by pulse radiolysis for reaction of Sc_3_N@C_80_(OH)_18_ with CCl_3_OO^•^ radicals is 1.29 × 10^7^ dm^3^ mol^−1^ s^−1^. The findings reveal that 25 µm Sc_3_N@C_80_(OH)_18_ protects human erythrocytes from radiation‐induced hemolysis. The protective effect is evidenced by reduced release of band 3 protein from radiation‐induced degradation up to 2.115 kGy. The observed effects are proposed to result from ROS scavenging by Sc_3_N@C_80_(OH)_18_ and its bioactivity via membrane protein adsorption. These findings highlight its potential for mitigating radiation‐induced membrane damage, consequently providing a promising foundation for further studies on application in, e.g., cancer therapy.

## Graphical abstract

Metallofullerenol Sc_3_N@C_80_(OH)_18_ demonstrates strong radioprotective properties as a scavenger of both short‐ and long‐lived radicals. The study reveals protection of human erythrocytes from γ‐radiation–induced biochemical damage via post‐irradiation removal of primary and secondary reactive oxidants, supported by pulse radiolysis kinetics. These findings highlight Sc_3_N@C_80_(OH)_18_ as a promising nanoplatform for cancer therapy and nuclear safety applications.

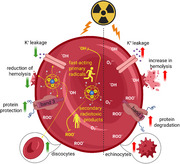

## 1 Introduction

The harmful effects of ionizing radiation (IR) are closely linked to elevated oxidative stress, resulting from the ionization of both organic and inorganic compounds.^[^ ^1^, ^2^ ^]^ The radiolysis of water stands out as the primary process contributing to the heightened formation of reactive oxygen species (ROS) in the cell.^[^ ^3^ ^]^ While these processes are important to the success of some medical interventions, e.g. modern cancer‐targeted radiotherapy, it is also crucial to minimize associated side effects for enhanced safety. Therefore, the identification of novel, safe, and efficacious nanocompounds capable of shielding healthy cells from IR‐induced damage is of paramount importance.^[^ ^4^, ^5^, ^6^, ^7^ ^]^ Guo et al.,^[^ ^6^ ^]^ recently reviewed the potential of nanodrugs in exerting radioprotection and showcased their pivotal role in preventing injuries due to nuclear accidents and radiation exposure. Fullerenes and their derivatives are promising compounds with great potential in biomedical sciences.^[^ ^8^, ^9^, ^10^, ^11^ ^]^ They can be used both in generating reactive oxygen species (ROS) for photodynamic therapy (PDT)^[^ ^12^ ^]^ and as ROS scavengers in radioprotection.^[^ ^13^, ^14^ ^]^ However, their mode of action depends on the experimental system used.^[^ ^15^ ^]^ Water‐soluble fullerenes and metallofullerene derivatives with hydroxy groups represent a new category of nanoparticles with strong antioxidant properties.^[^ ^16^, ^17^, ^18^ ^]^ These compounds are considered potential candidates for radioprotectors, offering significant advantages over conventional agents.^[^ ^13^, ^17^, ^19^, ^20^, ^21^ ^]^

Metallofullerenols (MFs) are synthesized by encapsulating diverse metal atoms or clusters within fullerene cages. Various MFs have been shown to be non‐toxic and well tolerated by biological systems up to high concentrations,^[^ ^18^, ^22^ ^]^ including in our previous studies on MFs with other encapsulated metal species,^[^ ^23^ ^]^ and they also exhibit favorable biological attributes, i.a. serving as free radical scavengers.^[^ ^14^, ^18^, ^19^, ^24^, ^25^ ^]^ In nanomedicine, gadolinium‐containing MFs have found application as magnetic resonance imaging contrast agents, signaling their medical utility. Potential medical applications of MFs extend beyond diagnostics and imaging.^[^ ^18^, ^26^, ^27^ ^]^ The protective capabilities of MFs against oxidative damage in various in vitro and in vivo cellular models may stem from the capacity for activating antioxidant and pro‐survival signaling pathways and transcription factors in response to sublethal doses of endogenous and exogenous ROS.^[^ ^14^, ^18^ ^]^ Recent findings indicate that MFs interact with the cell membranes of human erythrocytes, safeguarding against destruction induced by peroxyl radicals.^[^ ^23^ ^]^ Results of pulse radiolysis experiments reveal the rapid reactivity of MFs with the primary products of radiolytic decomposition of water. The rate constants for the reactions with *e*_aq_^−^ and the radical HO^•^ depend on the type of metal (or metal group) forming the cluster within the hydroxylated fullerene cage.^[^ ^19^ ^]^ Inspired by these observations, we assume that MFs hold significant promise as innovative radioprotective agents, with reactivity surpassing that of other fullerenes and fullerenols due to the presence of rare earth metal elements. Radical trapping efficiency of MFs combined with their relatively high polarity (leading to higher bioavailability) is a unique feature that enhances potential for inhibition of oxidative damage resulting from radiotherapy. The preferential localization of MFs at the lipid/water interface suggests a high degree of selectivity for biological membranes,^[^ ^23^ ^]^ providing a crucial advantage over other radioprotective agents.

Erythrocytes serve as a valuable and practical model cellular system for investigating the antioxidant mechanisms of small molecules and nanoparticles.^[^ ^16^, ^23^, ^28^, ^29^ ^]^ Their resemblance to living nucleated cells, with features such as a membrane composed of lipids and proteins (enzymes), as well as a biochemically active cytosol, makes them suitable for studying oxidative stress and the antioxidant effects of tested exogenous compounds. Notably, the absence of a cell nucleus and most organelles simplifies the monitoring of oxidative stress induction and facilitates the interpretation of results compared to other cell types. This is crucial, especially when distinguishing direct antioxidant action from potential indirect antioxidant effects from gene‐mediated adaptation in nucleated cells.^[^ ^1^, ^30^ ^]^ Erythrocytes also serve as a biophysical model for investigating membrane changes influenced by ionizing radiation.^[^ ^13^, ^19^ ^]^

## 2 Results

In our study, erythrocytes were intentionally selected as a model system because they are enucleated cells that do not undergo apoptosis. This unique property allows us to focus exclusively on radical‐induced damage and membrane‐related processes without interference from nuclear‐mediated adaptive responses, such as changes in gene expression. This feature of the erythrocyte model represents a key advantage in the present context, as it enables direct interpretation of the protective effects of metallofullerenols at the level of membrane integrity and oxidative damage to membrane components, which occur rapidly after irradiation. By using this approach, we can specifically study immediate and indirect biochemical consequences of radical‐mediated injury, while avoiding the complexities introduced by long‐term apoptotic pathways involving nuclear or mitochondrial signaling.

In order to select the optimal concentration of metallofullerenol Sc‐FulOH for studies on the mechanism of its radioprotective action, we performed two preliminary experiments. In vitro cultured cell lines were used to validate the range where Sc‐FulOH is not cytotoxic (nucleated cells are more susceptible to potentially damaging effects than erythrocytes) **Table** S1 (Supporting Information). Post‐irradiation hemolysis assay (a simple parameter that indicates the severity of damage to the integrity of cell plasma membranes induced by radiation) was used to test the concentration dependence of radioprotective effect (**Figure** S1, Supporting Information). Together, these results led to the selection of 25 µM Sc‐FulOH as a non‐toxic concentration with a very strong protective effect.

The hemolysis assay was further applied for a mechanistic investigation into the inhibition of gamma radiation‐induced damage in human erythrocytes by Sc‐FulOH. Cells were divided into three experimental groups: one that was irradiated without the presence of Sc‐FulOH, one to which Sc‐FulOH was added before irradiation, and a third group with Sc‐FulOH added immediately after irradiation. Results are shown in **Table** 1. The percentage of hemolysis was measured just after irradiation as well as after 1 h of incubation. We focused on the 60‐min window as the most reliable time point to assess the protective effect, since longer incubation involves unrelated irreversible processes (e.g., oxygen involvement, enzymatic degradation, progressive hemolysis) that obscure the initial radiation‐induced damage. Immediately after exposure, there was no increase in the percentage of hemolysis in any of the conditions, even at the highest radiation dose (2.115 kGy). After 1 h incubation, the percentage of hemolysis in cells untreated with Sc‐FulOH was significantly higher than in the samples containing Sc‐FulOH and exposed to 0.660 kGy and 2.115 kGy. When irradiated with the highest dose, more than 70% of hemoglobin was released from cells after 1 h of incubation. In contrast, when cells were irradiated in the presence of 25 µm Sc‐FulOH, after 1 h post‐radiation delay, there was no significant increase in hemolysis compared to control, non‐irradiated cells. When Sc‐FulOH was added to cells just after irradiation, it completely prevented post‐radiation damage to cells exposed to 0.660 kGy and strongly reduced the damage from the highest dose (only ≈17% of hemoglobin was released in this case). Thus, Sc‐FulOH shifts the threshold of hemolytic effect of radiation to much higher doses (even higher when preincubated with erythrocytes prior to irradiation), and at the same time is able to mitigate the extent of damage at the highest dose (even if added after irradiation). These results demonstrate that Sc‐FulOH effectively protects the erythrocytes against the radiation‐induced hemolysis, but is less effective when Sc‐FulOH is added to the erythrocyte suspension directly after irradiation.

**Table 1:** Hemolysis of erythrocytes after irradiation in the presence or absence of Sc‐FulOH. ^*^ *p* < 0.05 (compared to 0 Gy); n = 3; #*p* <0.05 (compared to sample w/o Sc‐FulOH). Photographic images of representative tubes with supernatants from the 2.115 kGy‐irradiated sample are shown for illustration purposes.

Dose [kGy]	0	0.268	0.660	2.115	Image of Tube with Supernatant
**0 min after irradiation**					
without Sc‐FulOH	1.56 ± 0.12	1.82 ± 0.18	1.68 ± 0.14	1.32 ± 0.19	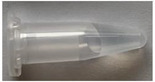
Sc‐FulOH before irradiation	1.68 ± 0.17	1.37 ± 0.09	1.70 ± 0.18	1.67 ± 0.17	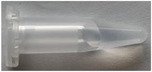
Sc‐FulOH after irradiation	1.86 ± 0.17	2.00 ± 0.18	1.91 ± 0.18	1.75 ± 0.17	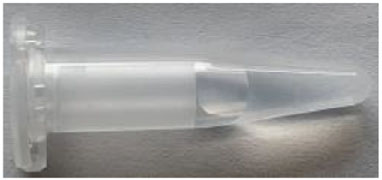
**60 min after irradiation**					
without Sc‐FulOH	1.79 ± 0.19	1.65 ± 0.13	7.33^*^ ± 0.14	73.03^*^ ± 0.31	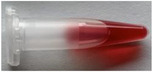
Sc‐FulOH before irradiation	2.05 ± 0.15	2.23 ± 0.18	2.20 ± 0.18	2.12^#^ ± 0.17	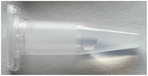
Sc‐FulOH after irradiation	2.32 ± 0.15	2.23 ± 0.21	2.01 ± 0.18	16.81^*#^ ± 0.27	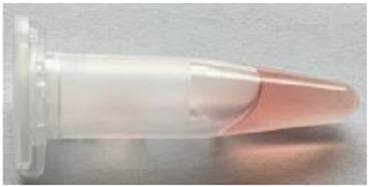

**Figure 1:**
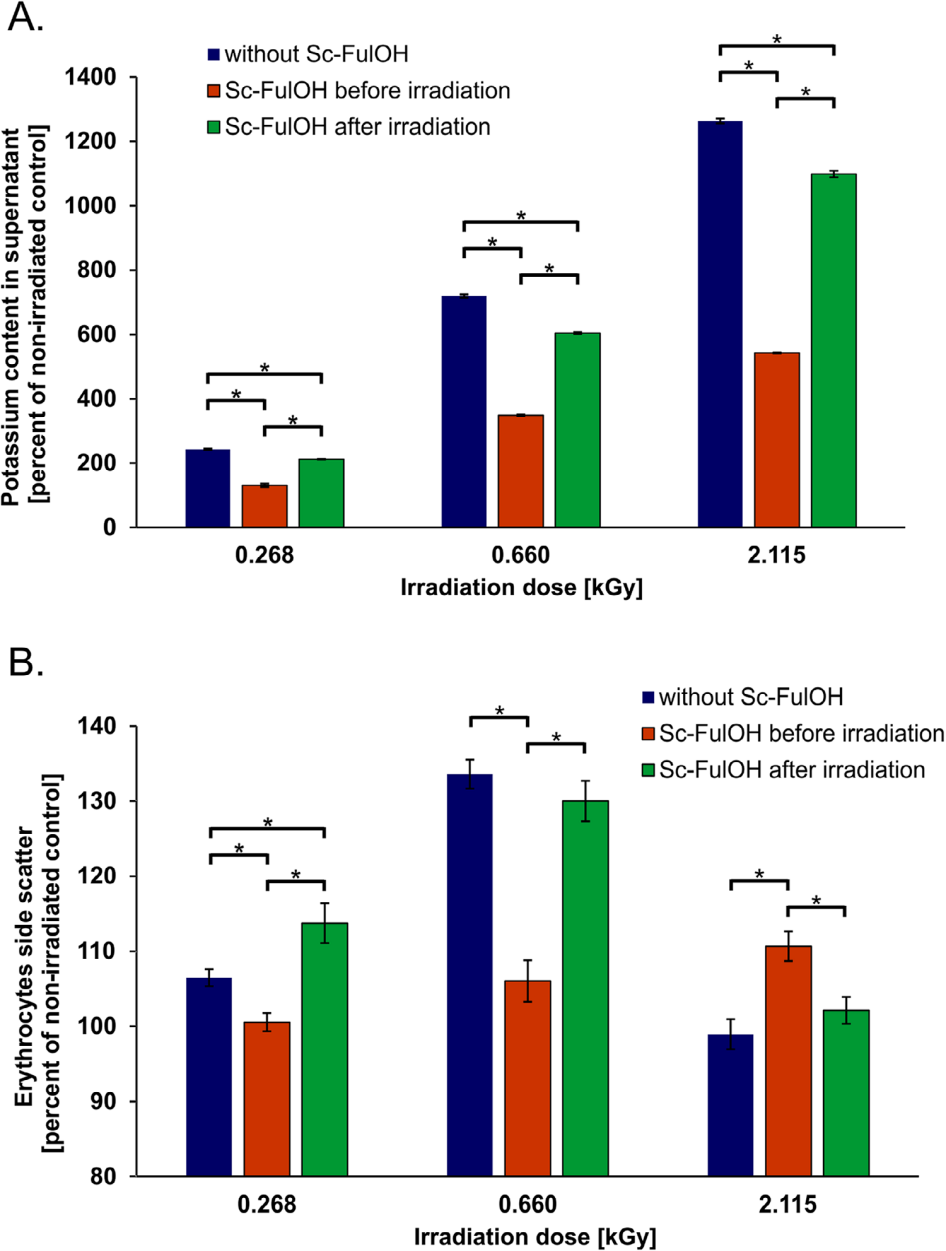
A) Release of potassium cation from erythrocytes upon irradiation in the presence or absence of Sc‐FulOH, n = 4, *p* <0.05. B) Changes in cell shape and granularity of erythrocytes upon irradiation in the presence or absence of Sc‐FulOH measured by flow cytometry side scatter, n = 4, ^*^ *p* <0.05.

Potassium release from erythrocytes was measured to directly assay transient, small‐scale damage to the membrane bilayer (ion leakage) as well as irreversible inhibition of sodium‐potassium ATPase activity over the period of irradiation (inhibition of ion re‐uptake). Data for the relative amount of released potassium is presented in **Figure** **1**A for samples irradiated: without Sc‐FulOH, after preincubation with Sc‐FulOH and with Sc‐FulOH added directly after irradiation. All radiation doses caused potassium leakage regardless of the presence of Sc‐FulOH, but the extent of membrane damage differed strongly, with ≈15% less potassium in the supernatant from erythrocytes treated after irradiation, decreasing down to ≈60% less potassium when treated before irradiation (at the highest dose of 2.115 kGy). This pattern of differences between Sc‐FulOH‐treated and ‐untreated samples can also be observed for lower doses, down to the lowest dose of 0.268 kGy. This observation is striking since the level of potassium in the supernatant was measured as soon as practicable after the end of the irradiation period, i.e., the time during which Sc‐FulOH could act in samples labeled as “Sc‐FulOH after irradiation” was practically limited to 5 min of centrifugation. Still, even such a short incubation was able to protect against more than 10% of the detrimental effects of radiation.

**Figure 2:**
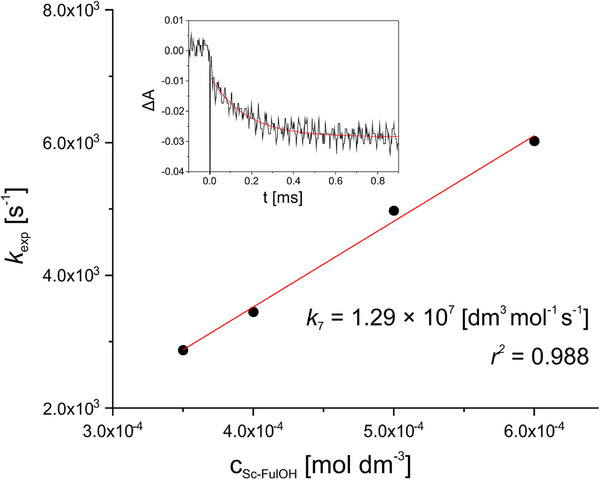
Values of pseudo‐first order rate constants for the reaction of CCl_3_OO^•^ + Sc‐FulOH plotted against increasing concentration of Sc‐FulOH (350–600 µm). *Inset*: typical decay of Sc‐FulOH monitored at 480 nm (initial concentration 600 µm) upon radiolytically generated CCl_3_OO^•^. The mechanism of generation of trichloromethylperoxyl radicals (equations (1), (2), (3), (4), (5), (6)) is presented in the Experimental section.

The above‐described results suggest that a significant mechanistic component of Sc‐FulOH radioprotective activity might happen just after the irradiation, involving reactions of Sc‐FulOH with primary products of radiolysis. We set out to verify whether this type of reaction is feasible in vitro, using model secondary radicals, i.e., peroxyls, generated in a sequence of reactions (1), (2), (3), (4), (5), (6), presented in the Experimental section. On the basis of kinetic analysis for the decay of Sc‐FulOH absorbance at 480 nm (see inset in **Figure** **2**), the pseudo‐first‐order rate constants *k*_exp_ (in s^−1^) were calculated for the reaction of radio‐generated CCl_3_OO^•^ radicals with an excess of Sc‐FulOH. Values of *k*_exp_ depend linearly on the concentration of the scavenger C_Sc‐FulOH_ (see **Figure** 2), and the slope of this straight line gives the bimolecular rate constant *k*_7_ = 1.29 × 10^7^ dm^3^ mol^−1^ s^−1^ for the reaction (7), making this reaction not only feasible, but relatively fast.

The structure of the erythrocyte membrane and its tight interaction with the cytoskeleton make the micro‐curvature of the membrane very sensitive to even slight disturbances in the physico‐chemical homeostasis of the membrane, including oxidative damage. Consequently, erythrocyte shape is a highly sensitive marker of membrane damage. We used lateral light scattering (SSC) measurement in a flow cytometer to detect modulation of shape in erythrocytes in all three experimental groups (**Figure** 1B). In erythrocytes untreated with Sc‐FulOH, two lower doses of radiation caused increasingly more severe aberrations of shape, while for the highest dose, the measured effect returned to the control value. These results must be interpreted with regard to the data presented in **Table** 1, which show very strong hemolysis in erythrocytes without Sc‐FulOH irradiated with the highest dose. At subhemolytic doses, gross integrity of the cell membrane is preserved, and damage to constituent lipids in individual leaflets produces the local and overall surface tensions, resulting in instability and shape changes. After the integrity of the membrane is compromised (in hemolyzed erythrocytes at the highest dose of radiation), these tensions are released and the shape of the resulting erythrocyte ghost reverts to normal, giving a remarkable biphasic effect of radiation in this assay, with a seemingly stronger impact of the lower dose than the higher one. This interpretation is strongly supported by the observed effect of Sc‐FulOH added after irradiation, which had a very weak effect on lateral light scattering, not decreasing the observable damage significantly even at the middle dose of 0.66 kGy. On the other hand, a sizeable protective effect was observed when Sc‐FulOH was present during irradiation, where for the lowest dose of radiation it completely prevented any changes in SSC, and at the middle dose it still had a very pronounced protective effect. At the highest dose, protection by Sc‐FulOH was masked by its ability to protect erythrocytes from hemolysis (as seen in **Table** 1), so the biphasicity of the dose‐dependence of SSC in the absence of protector is replaced by a continuous dose‐dependent increase of damage. Still, the modulation of erythrocyte shape by the highest radiation was much weaker in the presence of Sc‐FulOH than the damage caused in unprotected erythrocytes by a three‐fold smaller dose, so we can infer the protective effect on this membrane facet even despite the hemolysis‐related experimental artefact. We can conclude that the radioprotective effect of Sc‐FulOH against membrane damage is multifactorial and extends over a broad range of radiation doses.

**Figure 3:**
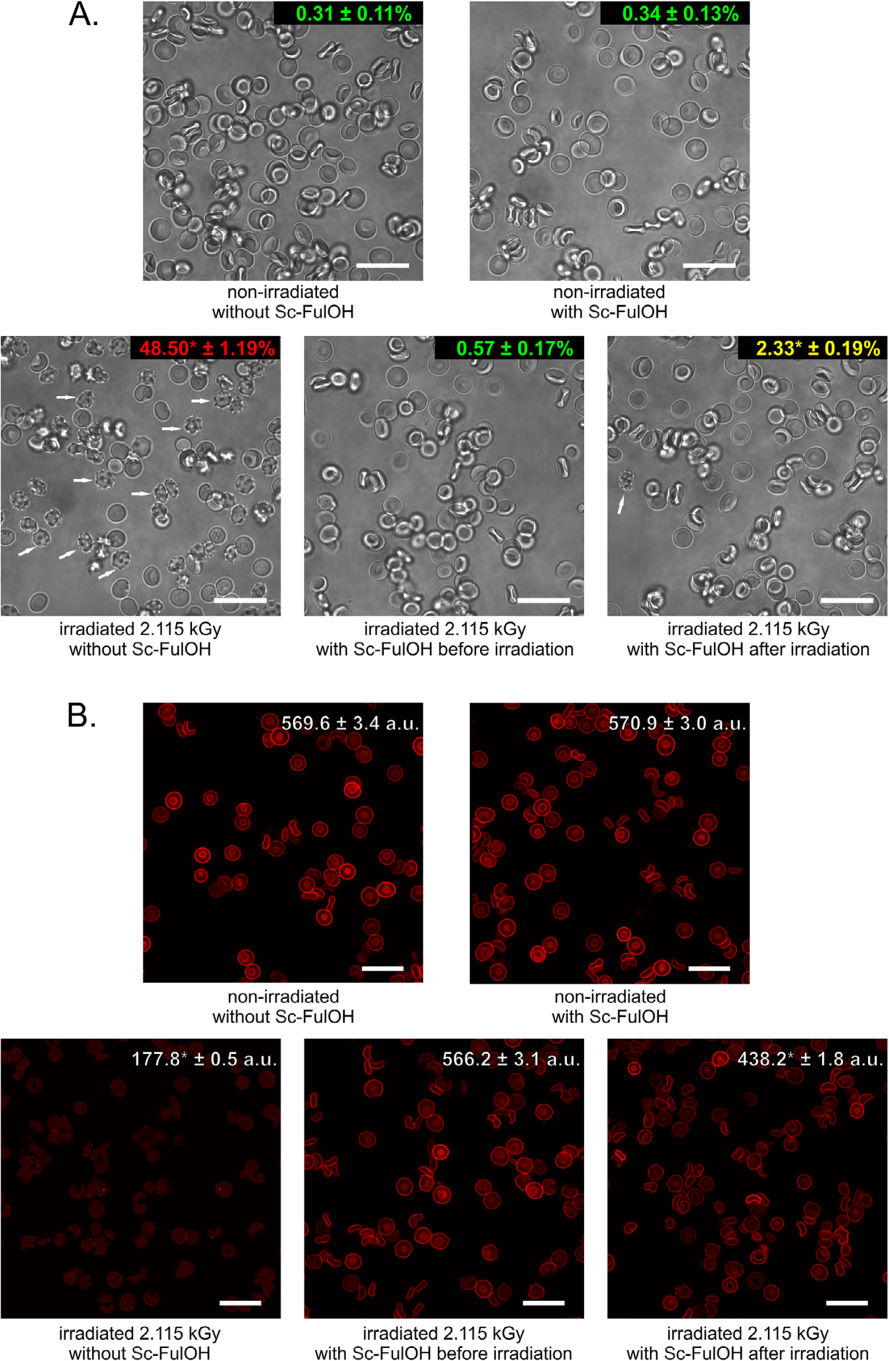
Microscopic analysis of changes in cell shape and expression of band 3 protein in erythrocytes upon irradiation in the presence or absence of Sc‐FulOH. Scale bars: 20 µm. A) Representative transmitted light images. Representative echinocytes are indicated with white arrows. Numbers shown on images describe the percentage of echinocytes present in the given sample, n = 4, ^*^ *p* <0.05. B) Representative images of erythrocytes with band 3 protein visualized by immunostaining. Cells were stained with primary anti‐band 3 antibody and secondary antibody conjugated with Alexa Fluor 594 (red signal). Numbers shown on images represent mean fluorescence intensity of pixels containing positive (above threshold) Alexa Fluor 594 fluorescence signal within individual cells in the given sample, n = 2000, ^*^ *p* <0.05 (compared to non‐irradiated cells without Sc‐FulOH).

Interpretation of changes in light scattering by erythrocytes (increase in cytometric SSC values) is even more complex by the fact that it may involve several types of impacts on membrane integrity: local protrusions on damaged membrane due to disrupted interactions with cytoskeleton, disruption of membrane asymmetry due to local short‐lived perforations, as well as changes in overall tension between membrane monolayers due to chemical modification of lipid side chains. We directly observed changes in overall cell shape using microscopy (**Figure** **3**), and it is clear that the radiation dose and time scale applied by us led to the formation of echinocytes, i.e., large‐scale shape modifications that reflect abnormalities in both cytoskeletal anchoring and inter‐leaflet lipid imbalance. Since echinocyte formation is a threshold phenomenon (occurring according to the all‐or‐nothing principle), the appropriate quantitative measure of membrane damage in this case is the overall percentage of echinocytes in the sample (presented as numbers in **Figure** 3A). Properties of biological membranes might depend on their direct interactions with fullerenols,^[^ ^35^, ^36^, ^37^ ^]^ however, we found that Sc‐FulOH had no effect on erythrocyte shape in control (non‐irradiated) samples. On the other hand, incubation with Sc‐FulOH resulted in strong inhibition of echinocyte formation: when added before irradiation, the protective effect was nearly complete (the percentage of echinocytes was only twice bigger than in non‐irradiated samples compared to 150‐fold increase observed for the sample irradiated without Sc‐FulOH). When Sc‐Ful‐OH was added after irradiation, the protection was weaker, but still significant (seven‐fold increase in echinocyte formation with regard to the non‐irradiated sample). This strongly supports a protective mechanism based on, or partially consisting of, the elimination of secondary reactive products.

Since cell shape changes may be caused not only by damage to the lipid bilayer, but also by disrupted protein‐protein interfaces between integral membrane proteins and the cytoskeleton, we tested the immunoreactivity of the chloride/bicarbonate anion exchanger (band 3 protein). Its decrease is a well‐established marker for radiation‐induced damage to membrane proteins. Confocal microscope images of immunostained erythrocytes allowed to measure the overall amount of bound antibody (shown as numbers in **Figure** 3B), which decreases if epitopes are lost, either by disruption of protein conformation or by proteolysis of the whole molecule (both can result from radiation‐induced oxidative damage). Presence of Sc‐FulOH during irradiation completely prevented the strong decrease in band 3 immunoreactivity observed in irradiated erythrocytes. Moreover, addition of Sc‐FulOH after irradiation also had a relatively strong protective effect on band 3 (even stronger than for previously described hemolysis or shape change experiments) – this shows that protein damage is predominantly caused by long‐term action of secondary reactive oxidants produced after irradiation.

**Figure 4:**
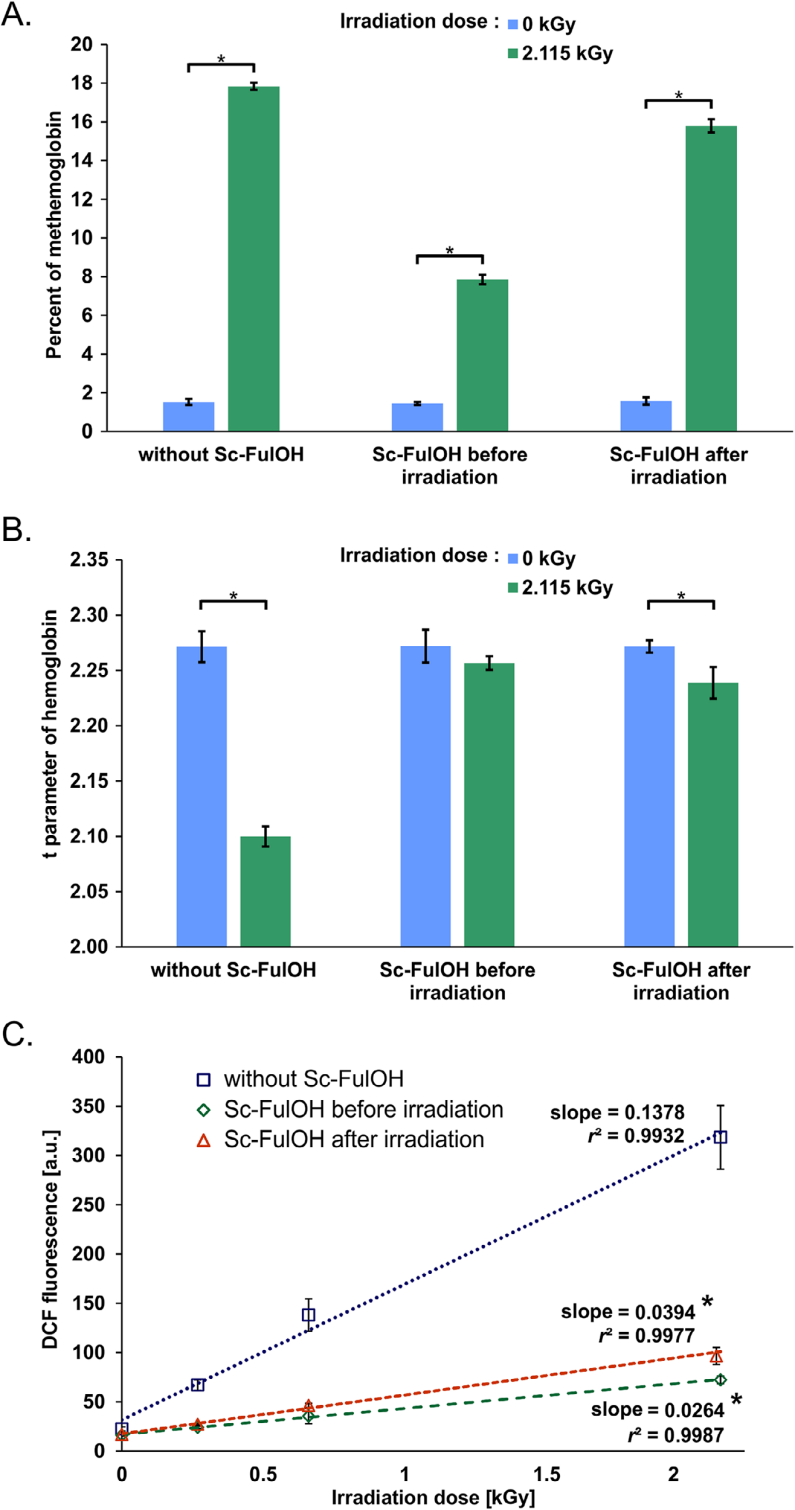
Changes in hemoglobin properties in erythrocytes upon irradiation in the presence or absence of Sc‐FulOH. A) Percent of hemoglobin oxidized to methemoglobin, n = 3, ^*^ *p* <0.05. B) Changes in *t* parameter, n = 3, ^*^ *p* <0.05. C) Accumulation of ROS measured by an increase in DCF fluorescence in erythrocytes upon irradiation in the presence or absence of Sc‐FulOH. Data represents average fluorescence intensity along with linear regression and respective slope and determination coefficient *r* ^2^ for each sample type. n = 4, ^*^ *p* <0.05 (compared to cells without Sc‐FulOH).

Heme proteins are also crucial targets of radiation damage, and changes in hemoglobin properties can be applied as a sensitive marker of direct and indirect effects of ionizing radiation. We measured two separate parameters: oxidation of heme iron (measured spectrophotometrically as percentage of methemoglobin) and the *t* parameter of globin structure (absorbance ratio measuring the bathochromic spectral shift stemming from globin polypeptide denaturation and disruption of the distance and interactions between alpha and beta chains). While methemoglobin formation can be mediated by various primary and secondary oxygen radicals and oxidants, the *t* parameter decreases only after very strong damage mediated by highly reactive irradiation‐derived molecules. Looking at those two parameters, we observed different patterns of protection by Sc‐FulOH. The strong effect of high‐dose gamma radiation on heme iron oxidation (**Figure** **4**A) was slightly decreased (by ≈12%) when Sc‐FulOH was added after irradiation. In contrast, remarkably better protection (decrease of ≈44%) was observed when Sc‐FulOH was present during irradiation, but even in this case Sc‐FulOH was not able to fully prevent the damage, reflecting the very quick and mostly primary character of this radiation‐induced process. Results for non‐irradiated samples show no difference in MetHb level in the absence and presence of Sc‐FulOH, show conclusively that protection is not due to any repair by direct electron transfer between Sc‐FulOH and methemoglobin, but the role of Sc‐FulOH is to quench reactive oxidants (both short‐lived and long‐lived ones, for example, peroxyl radical) generated during irradiation. In contrast, the extent of protective action of Sc‐FulOH was significantly larger for the *t* parameter (**Figure** 4B). Even when added after irradiation, Sc‐FulOH was able to prevent denaturation of globin chains by more than 80%. When Sc‐FulOH was present during irradiation, only a background amount of damage appeared, demonstrating a full protection. This means that chemical mediators of polypeptide denaturation, as more reactive, are easier to scavenge by Sc‐FulOH than the chemical entities oxidizing Fe^2+^ in heme.

The observed ability of Sc‐FulOH to alleviate radiation‐induced damage of cellular (biological) components prompted us to measure the direct impact of Sc‐FulOH on the concentration of intracellular ROS generated by radiolysis. Experiments with DCFH_2_ indicated a linear dependence of ROS concentration on ionizing radiation dose in this relatively simple biological material (**Figure** 4C). Sc‐FulOH is a highly efficient ROS scavenger in irradiated erythrocytes (ROS depleted by more than 80%), and almost the same efficiency was observed when Sc‐FulOH was added after irradiation as when it was present during irradiation. This observation proves that Sc‐FulOH is a very efficient scavenging radioprotectant, capable of removing not only highly active ROS but also other species having a relatively long lifetime but able to oxidize the fluorogenic probe (as well as important cellular components), and capable of conferring delayed radiodamage.

## 3 Discussion

Protection of biological organisms and tissues against the damaging effects of ionizing radiation is an important practical task and a real challenge. Radioprotectants that work efficiently in biological systems without deleterious side effects are rare, and very eagerly awaited by medicine and by industry.^[^ ^2^, ^7^, ^38^, ^39^ ^]^ Radioprotectants are needed in many fields where there is a need to protect humans or other living beings from radiation, and limitation of exposure is impractical or not sufficient. Despite great improvement in the design of irradiation equipment used in radiation therapy, it is impossible to avoid irradiating healthy tissues.^[^ ^7^, ^40^, ^41^ ^]^ Apart from clinical treatment, radioprotection is of crucial importance for example, after accidental overexposure in radiosterilization, especially of tissue for transplantation,^[^ ^42^ ^]^ protection of rescue groups acting during nuclear emergencies,^[^ ^43^, ^44^, ^45^ ^]^ and quite pressingly, in crewed space exploration, which always exposes astronauts to cosmic radiation.^[^ ^46^, ^47^ ^]^

Unfortunately, currently used radioprotectants are unsuitable for at least some of these applications due to their modes of action, even those that still remain at early research stages. Amifostine, the radioprotectant most commonly used in clinical settings, locally scavenges highly reactive radicals, but its pharmacokinetics do not allow for long‐term action, it is ineffective against some less reactive secondary radiochemical products, and the requirement for its presence during irradiation itself limits its application.^[^ ^48^, ^49^ ^]^ Amifostine and other commonly used compounds with similar modes of action (e.g., N‐acetylcysteine) must be applied at relatively high doses and thus can sometimes have very severe side effects.^[^ ^2^, ^50^ ^]^ In studies using erythrocytes as radiation damage models, amifostine has been shown to have lower protective efficiency than that seen in our research due to its reliance on phosphatase activation;^[^ ^51^ ^]^ similarly strong protection against short‐term acting oxidants was demonstrated by the vitamin E analog Trolox.^[^ ^52^ ^]^

Alternative mechanisms of pharmacological radioprotection involve: restoration of tissue by stimulating cell proliferation rather than actually protecting against damage, for example G‐CSF analogs (like filgrastim);^[^ ^2^, ^53^, ^54^ ^]^ triggering the expression of anti‐oxidant and gene repair enzymes, e.g. by stimulating the NF‐kappaB pathway (entolimod);^[^ ^55^ ^]^ chelation of radionuclides and transition metals responsible for Fenton‐type reactions that exacerbate oxidative damage (e.g., metformin or diethylenetriaminepentaacetic acid);^[^ ^56^, ^57^ ^]^ stopping cell division to prevent chromosomal damage during mitosis (e.g., genistein);^[^ ^58^ ^]^ or stimulating pro‐survival signaling pathways leading to DNA repair rather than apoptosis (recilisib).^[^ ^59^, ^60^ ^]^ These mechanisms are not versatile, have intrinsically high potential for side effects, are difficult to apply efficiently in a practical setting, and may interfere with other pharmacological treatments (e.g., stimulation of survival may conflict with anti‐cancer treatment).

Any functional biological radioprotectant should scavenge ROS which are responsible for most adverse effects of radiation exposure at the biophysical, biochemical, and ultimately cellular level. However, scavenging exclusively the primary radicals derived from radiolysis (especially water radiolysis) would be an inefficient mode of protection. These radicals are extremely reactive and short‐lived – they could never be removed quickly enough or completely enough from biological systems by any scavenger, because its concentration would have to exceed the concentrations of most biomolecules to protect them. Moreover, most of the actual biological damage from irradiation is caused by longer‐lived, secondary products that inflict relatively slow‐acting damage to constituent macromolecules in cells (proteins, lipids, nucleic acids).^[^ ^61^, ^62^ ^]^ A potent radioprotectant should thus protect these macromolecules (and consequently organelles and cells) during minutes and hours after irradiation, when most damage takes place in locations unaffected by primary radicals. It should also scavenge carbon‐ or oxygen‐centered^[^ ^5^ ^]^ secondary radicals as well as ternary molecular damage products, which often lead to damaging chain reactions in biological systems.

Our research demonstrated that Sc‐FulOH fulfils the described requirements well. A crucial feature is the capacity to alleviate damage even when not present during the immediate interaction between radiation and the biological system. We postulate that the reason for this advantageous property is its fast reaction rate with short‐lived and long‐lived radicals.^[^ ^19^ ^]^ A salient example is the fast reaction of Sc‐FulOH with organic peroxyl radicals. Obtained in this work value *k*_7_ = 1.29 × 10^7^ dm^3^ mol^−1^ s^−1^ is much higher than typical rate constant for reaction of CCl_3_OO^•^ with phenols and comparable to values within the range (0.9‐4.6) × 10^7^ dm^3^ mol^−1^ s^−1^ reported for reaction of CCl_3_OO^•^ with Trolox (analog of α‐tocopherol) in CCl_4_, acetone, and isopropanol, i.e., the same solvents as in our experiments.^[^ ^63^ ^]^ Serendipitously, Sc‐FulOH is not reactive toward most normal cell constituents, making it practically bioorthogonal at the concentrations needed for radioprotective action.^[^ ^19^, ^22^ ^]^ Fullerenes are known as efficient free radical scavengers due to high density of delocalized *π* electrons, but their extreme hydrophobicity prevents their direct use in biological systems.^[^ ^64^, ^65^ ^]^ Hydroxylation renders fullerenes more water‐soluble and turns them into practically effective radioprotectants, even in vivo, although protective efficiency is suboptimal.^[^ ^1^, ^13^, ^15^, ^66^, ^67^, ^68^, ^69^ ^]^ Addition of endohedral metal compounds to fullerenes is known to increase the electron affinity of the carbon core, making it easier to undergo redox reactions.^[^ ^70^ ^]^ A theoretical study on a lanthanum metallofullerenol demonstrated synergy between the presence of metal and the number of hydroxy groups for increased electron donor potency.^[^ ^71^ ^]^ This is the reason why inclusion of metal atoms (e.g., gadolinium)^[^ ^24^, ^72^, ^73^ ^]^ or metal nitrides (scandium nitride in the present study) increases antioxidant properties of fullerenols.^[^ ^19^ ^]^ Thus, Sc‐FulOH represents the culmination of long‐term research into optimization of the fullerene structure as a radioprotective agent for biological systems, and the impressive effects seen in the present study vindicate this direction of investigations.

Notably, Sc_3_N@C_80_ the precursor of Sc‐FulOH — was identified in another study as exhibiting a particularly shallow minimum in anion yield and a maximum shifted toward lower electron energies compared to other congeners.^[^ ^74^ ^]^ We confirm this pattern and extend it to redox chemistry of this compound: for example, the rate constant of 2.0 × 10^6^ dm^3^ mol^−1^ s^−1^ obtained by Dimitrijević^[^ ^75^ ^]^ for the reaction of (trichloromethyl)peroxyl radical with unmodified C_60_ fullerene is 6.5‐fold lower than the rate constant determined in this work for the same radical reacting with Sc‐FulOH, confirming the stronger reducing potency of the latter. The most probable course of radical trapping is its addition (as a whole) to an unsubstituted sp^2^ carbon atom in the Sc‐FulOH shell. We postulate that the same mechanism is valid for Sc‐FulOH reducing the lipid peroxyl radicals formed upon biological membrane irradiation in our erythrocyte model, since trichloromethylperoxyl radical is an established, highly equivalent analog of lipid peroxyls.^[^ ^76^ ^]^ One‐electron reduction potential of CCl_3_OO^•^ is higher than for typical lipid‐derived peroxyl radicals (1.3 vs 1.0 V).^[^ ^76^, ^77^ ^]^ With this in mind, lipid peroxyl radicals should be even less prone to simple electron capture reduction, with adduct formation as the favored mechanism instead. A similar mechanism was postulated for the addition of organic radicals to double bonds in graphene oxide,^[^ ^78^ ^]^ but this compound is unsuitable for application in biological systems.

Comprehensive selection of erythrocyte‐specific radiation damage biomarkers (relevant to the main goal of demonstrating radioprotection against membrane damage), together with datapoints where Sc‐FulOH was added at different moments in the experiment, allowed us to demonstrate the dual character of radioprotective action of Sc‐FulOH. On one hand, when present throughout the experiment (i.e., also during the relatively long process of irradiation), Sc‐FulOH had a strong protective effect for erythrocyte biochemistry and physiology in every damage endpoint we studied, in some cases entirely reverting observed radiodamage to control levels. On the other hand, when Sc‐FulOH was added immediately after the irradiation, we could distinguish two distinct types of response. In measurements of hemolysis, echinocytosis, band 3 protein degradation, globin denaturation, and ROS accumulation, the protective effect was also quite strong, often nearly as strong as when Sc‐FulOH was present from the beginning (“type ii” response). In measurements of potassium leakage, cell membrane granularity, and heme oxidation when Sc‐FulOH was added after the irradiation, the protective effect was very weak and damage was almost as extensive as without any protectant, even for large radiation doses (“type i” response).

It is known that damage biomarkers with type i response are a consequence of cell injury, which occurs very fast, usually caused by highly reactive (short‐lived) factors.^[^ ^34^, ^79^, ^80^ ^]^ However, this damage is often reversible and does not always lead to grave consequences for the cell as a whole. Specifically, potassium leakage is observed either due to the formation of minute, transient permeability pores in the bilayer which reseal spontaneously,^[^ ^81^ ^]^ or due to transient inhibition of the sodium‐potassium pump responsible for maintaining potassium gradient across the cell membrane.^[^ ^82^ ^]^ Increased lateral light scattering is caused by optical incongruencies in membrane structure ‐ localized membrane wrinkles caused by lipid imbalance between bilayers^[^ ^83^, ^84^ ^]^ or point detachment from the underlying cytoskeleton.^[^ ^36^, ^84^, ^85^ ^]^ Iron in hemoglobin can be partially oxidized even in physiological conditions^[^ ^86^ ^]^ and can be reduced back to the Fe^2+^ state by cytochrome‐*b*5 reductase (EC 1.6.2.2), an enzyme present in human erythrocytes.^[^ ^87^, ^88^ ^]^ When the offending factor (here: radiation) is removed after a sufficiently short time of action, these types of damage stop progressing immediately (since they are caused mainly by highly reactive free radicals ‐ direct radiolysis products^[^ ^19^, ^89^, ^90^ ^]^) and partially or completely revert due to spontaneous biological repair.

Conversely, biomarkers which show a type ii response in our experiments correspond to cell damage that is long‐lasting, caused directly by long‐lived, secondary products of oxidative reactions, and usually difficult to efficiently repair in a biological system without genetically regulated protein biogenesis (which erythrocytes completely lack). Specifically, hemolysis is the erythrocyte equivalent of necrotic cell death as a full and irreversible loss of cell membrane integrity and internal content.^[^ ^16^, ^91^ ^]^ Echinocytosis is caused by a conjunction of multi‐point loss of cytoskeletal structure and generalized irregularities in lipid asymmetry between membrane leaflets.^[^ ^83^, ^84^ ^]^ Loss of tertiary and secondary protein structure as well as proteolytic degradation (in our experiments measured for band 3 protein) is irreversible in erythrocytes.^[^ ^92^, ^93^ ^]^ The DCFH_2_ probe is responsive mainly to long‐lived reactive oxygen species that have the propensity to accumulate in cells and cause progressive oxidative damage to macromolecules. These ROS include superoxide, hydrogen peroxide, and peroxynitrite^[^ ^94^, ^95^ ^]^ and are known to be jointly responsible for most of the secondary radiotoxicity in erythrocytes.^[^ ^13^, ^19^ ^]^

**Figure 5:**
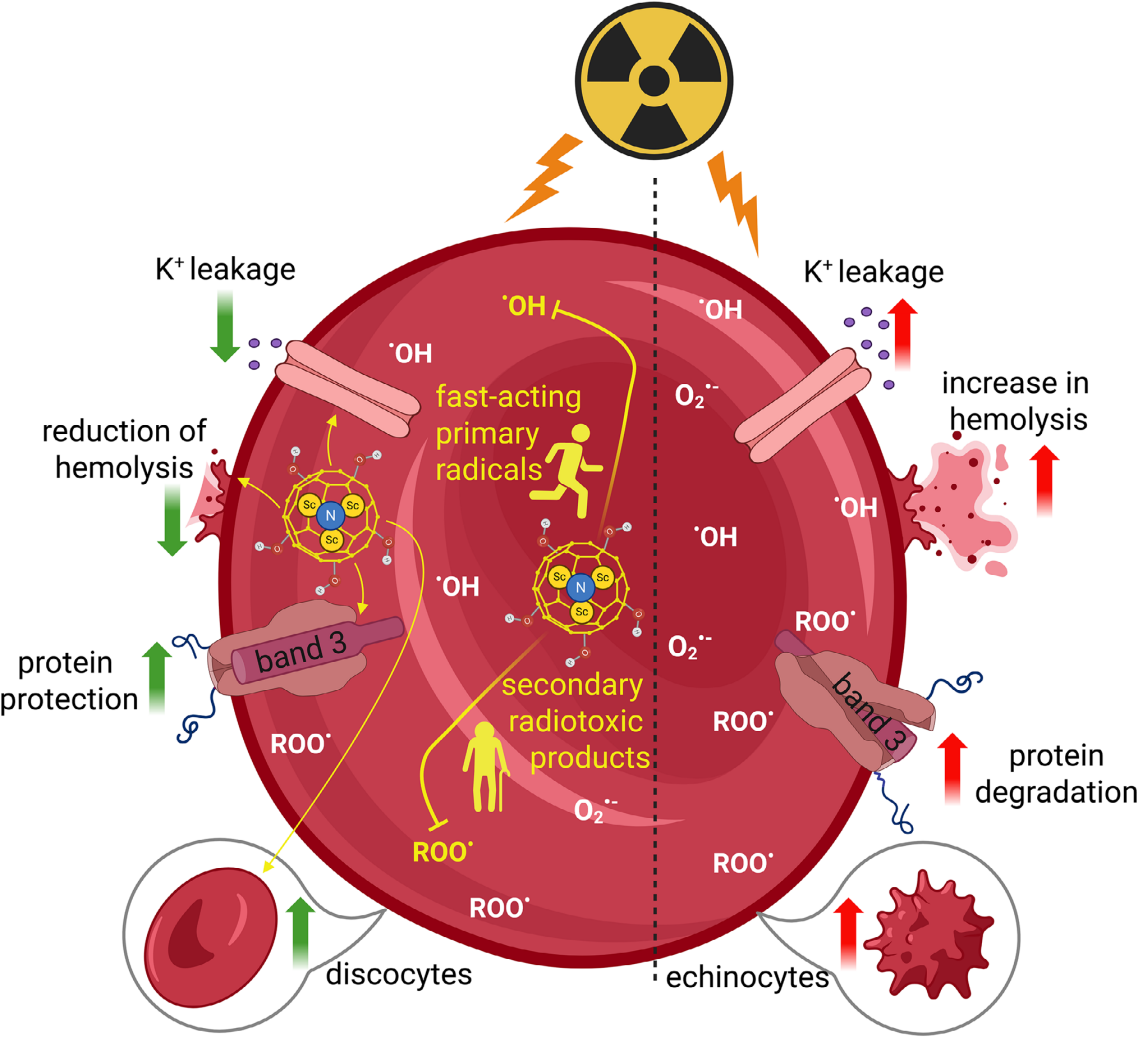
Dual‐mode radioprotective mechanism of metallofullerenol Sc_3_N@C_80_(OH)_18_ (Sc‐FulOH) in human erythrocytes exposed to gamma irradiation. Sc‐FulOH scavenges short‐lived primary radicals during irradiation, effectively preventing type i damage, including potassium ion leakage, changes in membrane granularity, and heme oxidation—lesions that are fast‐occurring and often partially reversible. When present during irradiation, it efficiently neutralizes these early lesions. Even when administered after irradiation, Sc‐FulOH mitigates type ii damage caused by long‐lived secondary radiotoxic products, protecting cells from largely irreversible effects such as hemolysis, echinocytosis, degradation of band 3 protein, globin denaturation, and accumulation of reactive oxygen species (ROS). This distinction highlights the dual and time‐dependent protective role of Sc‐FulOH in minimizing both early, transient and late, persistent biochemical damage in irradiated erythrocytes.

In summary, this body of data clearly suggests a dual mode of action for Sc‐FulOH: it is capable of scavenging fast‐acting primary radicals responsible for type i damage biomarkers (only when present during their generation in the process of irradiation); it is also capable of efficiently preventing more dangerous, irreversible type ii damage caused by secondary radiotoxic products (even when added after irradiation, see **Figure** **5**)

This means that its practical use may also have a dual capacity: capability of entering the chemical reactions needed for long‐term radioprotection would determine its efficacy as emergency radioprotectant for already exposed individuals/tissues, but its even higher efficiency when present during irradiation (to scavenge the short‐lived radicals upon their generation) would warrant its use as preventive radioprotectant, administered before risk of exposure. This consistent pattern seen in our essays, with protection against chronic (and often strongly cytotoxic) damage exerted by Sc‐FulOH added after irradiation, demonstrates a high versatility of this radioprotectant.

Obviously, for any practical application, a successful radioprotectant must not only be effective but also biologically safe. Fullerenols are well known for their bioorthogonality, low toxicity, and lack of deleterious interactions with major enzymes and cellular components.^[^ ^16^, ^96^ ^]^ Moreover, their rigid, shell‐like structure forms a stable cage for embedded metal compounds, protecting against inadvertent leakage and toxicity of metal ions. For Sc‐FulOH, this potential risk is further mitigated by the fact that scandium has one of the lowest toxicities among transition metals: LD_50_ for oral administration of scandium chloride to rats is 4 g kg^−1^.^[^ ^97^ ^]^ In any case, spectral analysis confirmed that Sc‐FulOH remains stable upon incubation and/or irradiation under conditions much harsher than those applied in the present study (**Figure** S2, Supporting Information). Even more promising for practical use, fullerenols are ideally poised for comprehensive pharmacokinetic distribution, being neither too hydrophobic nor too hydrophilic, thus being able to access membrane compartments and scavenge lipid radicals from them, to distribute quickly throughout the body due to their good water solubility, and to traverse physiological barriers.^[^ ^98^ ^]^ The number of applications for a potentially effective and safe radioprotective agent is large and still growing. The high number of biological endpoints and detection modalities allows us to make well‐founded conclusions about the potential utility of Sc‐FulOH as a practical candidate molecule for preventive and emergency radioprotection. Although our data was obtained using a relatively simple model of a non‐nucleated cell, and we cannot be sure whether it would behave as potently in complex tissues, organs, and organisms, the promise is strong. Therefore, it is necessary (and in our immediate field of interest) to plan pre‐clinical and clinical experiments to fully uncover the impressive potential of this promising compound, discovered in the present study.

## 4 Experimental Section

### Chemicals

Sc_3_N@C_80_ was purchased from SES Research (Houston, TX, USA). Benzene, dialysis bags (MWCO = 1000 Da), and methanol were purchased from Sigma–Aldrich (St. Louis, MO, USA). Sodium hydroxide and toluene were purchased from POCh (Gliwice, Poland). All solutions were made with deionized water purified by the Milli‐Q system.

### Metallofullerenol Synthesis

The metallofullerenol Sc_3_N@C_80_(OH)_18_ abbreviated elsewhere as Sc‐FulOH was synthesized using the method reported by Mikawa et al.,^[^ ^26^ ^]^ in a modified version as described in the previous paper.^[^ ^19^ ^]^ The characteristics of Sc‐FulOH (infrared, thermogravimetric measurements, dynamic light scattering, and X‐ray photoelectron spectroscopy) as well as the methodology of its purification are described in our previous article.^[^ ^19^ ^]^ We have previously shown that Sc‐FulOH synthesized in this way shows 100% metal encapsulation and very high cage stability preventing any metal leakage.^[^ ^19^ ^]^ Sc‐FulOH stock solution (500 µm) was prepared in deionized water and sonicated for 10 min immediately before adding to the sample (final concentration 25 µm).

### Pulse Radiolysis Measurements

Trichloromethylperoxyl radicals CCl_3_OO^•^ were generated in aqueous solution saturated with oxygen containing carbon tetrachloride (10 mm), 2‐propanol (2 m), and acetone (1 m) which was irradiated with an electron beam. Electron pulses were delivered from the linear accelerator (LINAC) ELU‐6E operating in a single pulse mode. Radiation‐generated transient species were monitored using the real‐time optical detection system. Details of the equipment are described elsewhere.^[^ ^31^ ^]^ The radiolysis of water produced three well‐characterized reactive radical species (HO^•^; H^•^; *e*_aq_^−^), as well as molecular products (H_2_O_2_; H_2_). Radiation chemical yields (the *G* value) of HO^•^, H^•,^ and *e*_aq_^−^ and are 2.9, 0.6, and 2.9, respectively (*G* species per 100 eV absorbed radiation energy).

CCl_3_OO^•^ radicals were generated according to the following reactions:^[^ ^32^ ^]^

(1) $$\begin{array}{ccc} & & {\mathrm{HO}}^{•}+{\left({\mathrm{CH}}_{3}\right)}_{2}\mathrm{CHOH}\to H_{2}O+{\left({\mathrm{CH}}_{3}\right)}_{2}C^{•}\mathrm{OH}\;\;\\ & & k_{1}=2.0\times 10^{9}{\mathrm{dm}}^{3}\mathrm{mo}l^{-1}s^{-1} \end{array}$$

(2) $$\begin{array}{ccc} & & H^{•}+{\left({\mathrm{CH}}_{3}\right)}_{2}\mathrm{CHOH}\to H_{2}+{\left({\mathrm{CH}}_{3}\right)}_{2}C^{•}\mathrm{OH}\;\;\;\\ & & k_{2}=8.7\times 10^{7}{\mathrm{dm}}^{3}\mathrm{mo}l^{-1}s^{-1} \end{array}$$

(3) $$\begin{array}{ccc} & & {e_{\mathrm{aq}}}^{-}+{\left({\mathrm{CH}}_{3}\right)}_{2}\mathrm{CO}\to {\left({\mathrm{CH}}_{3}\right)}_{2}{\mathrm{CO}}^{•-}\;\overset{+H^{+}}{⇌}{\left({\mathrm{CH}}_{3}\right)}_{2}C^{•}\mathrm{OH}\;\;\;\\ & & k_{3}=5.9\times 10^{9}\;{\mathrm{dm}}^{3}\mathrm{mo}l^{-1}s^{-1} \end{array}$$

(4)

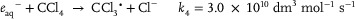

(5) $$\begin{array}{ccc} & & \mathrm{CC}l_{4}+{\left({\mathrm{CH}}_{3}\right)}_{2}C^{•}\mathrm{OH}\to {\left({\mathrm{CH}}_{3}\right)}_{2}\mathrm{CO}+\mathrm{CC}{l_{3}}^{•}+H^{+}+{\mathrm{Cl}}^{-}\;\;\;\\ & & k_{5}=7.0\times 10^{8}\;{\mathrm{dm}}^{3}\mathrm{mo}l^{-1}s^{-1} \end{array}$$

(6)

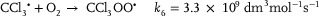




Processes 1‐6 are very fast, and taking into account high concentrations of isopropanol and acetone (above 1 m) the reactions 1‐2 eliminate HO^•^ and H^•^, and reaction 3 eliminates *e*_aq_^−^. Therefore, CCl_3_OO^•^ radicals are mainly formed through the subsequent reactions (5) and (6). Reaction (5) is the slowest, and this will be the rate‐controlling reaction. The total yield of CCl_3_OO^•^ can be estimated as *G* 4.8 ± 0.3 in this system. While isopropanol, acetone, and CCl_4_ were all present in large excess, Sc‐FulOH added at micromolar concentrations is not a competitive scavenger of HO^•^, H^•,^ and *e*_aq_^−^; however, peroxyl radicals are 10^3^ times less reactive and do not efficiently react with the solvents, but they can react with Sc‐FulOH (reaction 7, studied in this work):

(7) $$\mathrm{CC}l_{3}{\mathrm{OO}}^{•}+\mathrm{Sc}-\mathrm{FulOH}\to \mathrm{products}\;\;k_{7}$$

### UV–Vis Spectral Analysis of Metallofullerenol Stability Under Irradiation

The stability of metallofullerenol solutions under high‐dose electron‐beam irradiation was monitored using UV–Vis spectroscopy (spectrophotometer Perkin Elmer Lambda 750). Samples were irradiated with the 6 MeV LINAC ELU‐6E operating in single‐pulse. After each irradiation pulse, the UV–Vis spectrum of the sample was recorded to detect any immediate spectral changes indicative of structural alteration or degradation of the metallofullerenol. Pulse parameters were chosen in the nanosecond domain (typical pulses: 17 ns, ≈55 Gy per pulse), with cumulative doses up to 6.6 kGy when required. Dose calibration for each series was performed using chemical dosimetry. To generate specific radical environments, solutions were preconditioned with the required additives prior to irradiation. Hydroxyl radicals (HO^•^) were generated by N_2_O saturation of aqueous solutions, converting hydrated electrons into HO^•^ (N_2_O + *e*_aq_^−^ + H_2_O → N_2_ + OH^−^ + HO^•^) to increase HO^•^ yield. Trichloromethylperoxyl radicals (CCl_3_OO^•^) were generated as described in Experimental Section (*Pulse Radiolysis Measurements*).

### Cytotoxicity of Sc‐FulOH

In the present study, four human cell lines were used: human lung fibroblasts MRC‐5, human fetal colon epithelial cells CCD‐841CoN, non‐tumorigenic mammary epithelial cells MCF‐10a, and immortalized human pancreatic ductal epithelial cells hTERT‐HPNE. All cell lines were purchased from the American Type Culture Collection (Manassas, VA, USA). MRC‐5 and CCD‐841CoN cells were maintained in Eagle's Minimum Essential Medium (EMEM) supplemented with 10% fetal bovine serum (FBS). MCF‐10a cells were cultured in MEBM (DMEM/F12 mixture) containing 5% horse serum, 20 ng mL^−1^ human recombinant epidermal growth factor (EGF), 10 µg mL^−1^ insulin, 0.5 µg mL^−1^ hydrocortisone, and 1% penicillin–streptomycin (100 U mL^−1^ penicillin, 100 µg mL^−1^ streptomycin). hTERT‐HPNE cells were maintained in medium consisting of 75% Dulbecco's Modified Eagle Medium (1 g L^−1^ glucose) and 25% Medium M3 Base, supplemented with 5% FBS, 10 ng mL^−1^ EGF, and 750 ng mL^−1^ puromycin. All cell cultures were incubated at 37 °C in a humidified atmosphere with 5% CO_2_. For cytotoxicity testing, cells were seeded in 96‐well plates at the following densities: MRC‐5, 5000 cells/well; CCD‐841CoN, 7000 cells/well; MCF‐10a, 5000 cells/well; and hTERT‐HPNE, 8000 cells/well. After 24 h, Sc‐FulOH was added at concentrations ranging from 0.001 to 80 µm for 72 h. To assess cell viability, the MTT assay was applied according to the method described previously.^[^ ^33^ ^]^ Cell viability was determined by comparing absorbance values of treated wells with those of untreated control wells (0 µm, set as 100%). Data are presented as mean ± SD from at least three independent experiments.

### Erythrocyte Preparation

Buffy coats were purchased from the Regional Center for Blood Donation and Transfusion in Lodz, Poland. Erythrocytes were separated by centrifugation at 2000 ×g for 10 min and washed twice with phosphate‐buffered saline (145 mm NaCl, 10 mm Na‐phosphate, pH 7.4). The cells were resuspended in the same buffer to a hematocrit of 2%. All procedures related to blood donation were carried out at the Regional Center for Blood Donation and Blood Treatment in Lodz, Poland. The blood donor recruitment was at the Center, according to national legal procedures and European Union regulations (including the regulation (EU) 2016/679 of the European Parliament and of the Council of April 27, 2016 on the protection of natural persons regarding the processing of personal data and on the free movement of such data).

### Treatment and Irradiation of Erythrocytes

A suspension of erythrocytes at a hematocrit of 2% in buffer (145 mm NaCl, 10 mm sodium phosphate buffer, pH = 7.4) was placed at various distances from a ^60^Co gamma radiation source and irradiated at room temperature under ambient air conditions for 40 min. This setup allowed for the application of different radiation doses: 0, 0.268, 0.660, and 2.115 kGy. The exact dose delivered to each sample was determined using an alanine dosimeter. Sc‐FulOH was added at a final concentration of 25 µm to selected erythrocyte samples either immediately before or immediately after irradiation. The radioactivity of the ^60^Co source was 42 kCi (1554 TBq).

### Hemoglobin Measurements

Immediately after irradiation or after 1 h incubation at 37 °C, 500 µl of erythrocyte suspension was centrifuged (2500 × g, 5 min) and the supernatant was collected (Hb_s). The resulting pellet of erythrocytes was suspended in 1.4 ml of distilled water to allow for complete hemolysis (Hb_p). Both Hb_s and Hb_p samples were treated with 100 mm (excess) K_3_[Fe(CN)]_6_ to fully convert hemoglobin into methemoglobin, and absorbance was measured at 630 nm. The percentage of hemolysis of erythrocytes H(%) was calculated as the ratio of hemoglobin released from the cells after irradiation to the total cellular Hb content considering the appropriate dilution factor (r):

(8) $$H\left(\%\right)=\frac{A_{630,\;\mathrm{Hb}\_s}}{A_{630,\;\mathrm{Hb}\_s}+\;r\cdot A_{630,\;\mathrm{Hb}\_p}}*100\%$$

The extent of globin chain denaturation was assessed as described previously^[^ ^34^ ^]^ by measuring the *t* parameter (*t* = A_500_/A_563_) for Hb_p samples completely oxidized with an excess of K_3_[Fe(CN)]_6_.

For hemoglobin oxidation measurement, erythrocytes were lyzed with digitonin (added to a final concentration of 0.1%) immediately after the irradiation. After centrifugation (14 000 x g, 10 min, 4 °C), the absorbance of oxidized hemoglobin (MetHb_s) was measured in the supernatant at 630 nm. Subsequently, an excess of K_3_[Fe(CN)]_6_ was added to the samples to fully convert hemoglobin into methemoglobin, and absorbance at 630 nm was measured again (MetHb_total). The percentage of methemoglobin was calculated by a ratio: A_630, MetHb_s_ /A_630, MetHb_total_.

### Measurement of Potassium Release

Immediately after irradiation, samples were centrifuged (2500 × g, 5 min) and the concentration of potassium ions was measured in the resulting supernatants by flame atomic emission spectrometry using a SpectrAA‐300 apparatus (Varian, Australia). An increase in potassium ion concentration was presented for each experimental point as a percentage of the value measured in the non‐irradiated control sample.

### Microscopic Imaging and Analysis

Irradiated erythrocytes were immediately fixed for 20 min with 0.05% glutaraldehyde diluted in PBS. Excess of the fixative was then quenched with 10% BSA in PBS, and erythrocytes were imaged directly in transmitted light (illumination with In Tune tunable diode laser set to 595 nm). Samples were imaged with an LSM 780 confocal microscope (Zeiss, Germany) equipped with a Plan‐Apochromat 63x/1.40 objective. Four images containing at least 1000 individual cells were analyzed, and the percentage of echinocytes was calculated for each sample. For visualization of band 3 protein, fixed cells were permeabilized and blocked with buffer A (10% normal goat serum; PBS, pH = 7.4; 0.2% Triton X‐100) for 1 h. Subsequently, cells were incubated overnight with primary anti‐band 3 antibody (NB120‐11012, Novus Biologicals, USA) diluted 1:1000 in buffer A and for 1 h with secondary goat anti‐mouse IgG2a antibody conjugated with Alexa Fluor 594 diluted 1:1000 in buffer A. Samples were imaged with a LSM 780 confocal microscope (Zeiss, Germany) equipped with Plan‐Apochromat 63x/1.40 objective. Multiple images were obtained in 600–733 nm emission range with excitation by In InTune tunable diode laser set to 595 nm. Average intensity of fluorescence (corresponding to intact band 3 protein level) was calculated in individual cells following image segmentation performed with ZEN software (ZEN 2.1 Blue, Zeiss).

### Cytometric Measurements

Irradiated erythrocytes were incubated at room temperature for 30 min with DCFH_2_DA added to a final concentration of 20 µm. DCFH_2_DA (Thermo Fisher Scientific, Waltham, MA, USA) is the diacetate of the fluorogenic probe DCFH_2_, the reduced form of dichlorofluorescein (DCF); DCFH_2_ is generated by intracellular deacetylation and subsequently can be oxidized to fluorescent DCF by reactive oxidants. Subsequently, flow cytometric analyses were performed on an LSRFortessa flow cytometer (Becton Dickinson, USA). FACSDiva software was used for acquisition and analysis. For each measurement, a minimum of 20 000 erythrocytes were analyzed in each condition (n = 4). Median values of lateral scattering peak area (SSC‐A) were averaged and expressed as a percentage of the non‐irradiated control. Median values of fluorescence intensity peak area were expressed in arbitrary units and used for linear regression calculation by the least squares method in Excel 365 (Microsoft, USA).

### Statistical Analysis

Multiple data point differences were tested with ANOVA and pairwise differences with Tukey's post‐hoc test. Data is presented as mean ± standard error of mean, all using the GraphPad 5.0 Software (La Jolla, CA).

## Conflict of Interest

The authors declare no conflict of interest.

## Author Contributions

J.G. contributed to the conceptualization, data curation, formal analysis, funding acquisition, investigation, methodology, project administration, resources, software, supervision, validation, visualization, writing of the original draft, and writing – review and editing. M.S. was involved in formal analysis, investigation, methodology, data curation, visualization, and writing – review and editing. S.L.‐P. contributed to the investigation. A.K. participated in the investigation, specifically in pulse radiolysis. M.W. was responsible for the investigation and formal analysis related to pulse radiolysis. G.L. contributed to formal analysis and methodology in pulse radiolysis, as well as writing, review, and editing. L.P. was involved in formal analysis, investigation, supervision, validation, writing of the original draft, writing ‐ review and editing.

## Supporting information



Supporting Information

## Data Availability

The data that support the findings of this study are available from the corresponding author upon reasonable request.
