# Supplementary material for: Metallofullerenol Sc3N@C80(OH)18: A New Generation Radioprotector Protecting Human Erythrocytes Against Multiple Biochemical Damage Modes Upon Gamma Irradiation, Identifying It as a Scavenger of Short‐ and Long‐Lived Radicals
Source: Adv Healthc Mater. 2025 Nov 13;15(7):e02621. doi: 10.1002/adhm.202502621 (PMC12908212; doi:10.1002/adhm.202502621)
Supplement: Supplementary file 1 — Supporting Information [file ADHM-15-0-s001.docx]

**SUPPORTING INFORMATION**

**Metallofullerenol Sc_3_N@C_80_(OH)_18_: A New Generation Radioprotector Protecting Human Erythrocytes Against Multiple Biochemical Damage Modes Upon Gamma Irradiation, Identifying It as a Scavenger of Short- and Long-Lived Radicals**

Jacek Grebowski^1,2*^, Maciej Studzian^1,5^, Szymon Lekki-Porebski^1^, Anna Konarska^3^,
Marian Wolszczak^3^, Grzegorz Litwinienko^4^, Lukasz Pulaski^1,5*^

^1^ Faculty of Biology and Environmental Protection, University of Lodz, Pomorska 141/143, 90-236 Lodz, Poland.

^2^ Military Institute of Medicine - National Research Institute, Szaserow 128, 04-141 Warsaw, Poland.

^3^ Institute of Applied Radiation Chemistry, Technical University of Lodz, Wroblewskiego 15, 93-590 Lodz, Poland

^4^ Faculty of Chemistry, University of Warsaw, Pasteura 1, 02-093 Warsaw, Poland

^5^ Laboratory of Transcriptional Regulation, Institute of Medical Biology PAS, Lodowa 106, 93-232 Lodz, Poland.

**KEYWORDS:**

metallofullerenol, oxidative damage, erythrocytes, radiotherapy, radioprotection

*Corresponding author:

Dr. Jacek Grebowski,

Faculty of Biology and Environmental Protection, Department of Oncobiology and Epigenetics, University of Lodz, Pomorska 141/143, 90-236, Lodz, Poland;

Military Institute of Medicine - National Research Institute, Szaserow 128, 04-141, Warsaw, Poland.

E-mail: [jacek.grebowski@biol.uni.lodz.pl](mailto:jacek.grebowski@biol.uni.lodz.pl) (J.G.)

**Table S1.** Cytotoxicity of Sc-FulOH in **MRC-5 – human lung fibroblasts,** **CCD-841CoN – human fetal colon cells**, **hTERT-HPNE – human pancreatic ductal cells** and **MCF-10a – human mammary epithelial cells**, across a range of concentrations. Results presented as mean ± SD.

| **viability  [% of control]** | **Sc-FulOH concentration [µM]** | | | | | | | | | |
| --- | --- | --- | --- | --- | --- | --- | --- | --- | --- | --- |
|  | **0.001** | **0.01** | **0.1** | **0.5** | **1** | **5** | **10** | **20** | **40** | **80** |
| **MRC-5** | 101.4 ±5.3 | 99.1 ±3.6 | 98.1 ±3.2 | 98.5 ±1.8 | 100.3 ±2.5 | 96.7 ±3.4 | 101.3 ±2.4 | 97.0 ±1.0 | 90.4 ±5.2 | 92.8 ±2.0 |
| **CCD-841CoN** | 100.2 ±1.2 | 98.0 ±1.0 | 99.6 ±2.9 | 95.9 ±3.8 | 94.1 ±4.4 | 93.3 ±8.1 | 92.5 ±6.2 | 99.6 ±8.3 | 96.4 ±10.2 | 93.2 ±5.3 |
| **hTERT-HPNE** | 99.2 ±4.6 | 99.9 ±2.8 | 99.9 ±1.0 | 97.9 ±1.9 | 98.4 ±1.5 | 100.9 ±3.5 | 101.5 ±4.5 | 103.3 ±2.2 | 99.2 ±2.7 | 95.4 ±3.4 |
| **MCF-10a** | 98.8 ±0.6 | 99.7 ±1.1 | 98.6 ±2.9 | 98.0 ±2.4 | 99.8 ±2.7 | 101.4 ±1.4 | 100.1 ±2.7 | 101.3 ±1.9 | 109.0 ±2.5 | 113.8 ±5.2 |


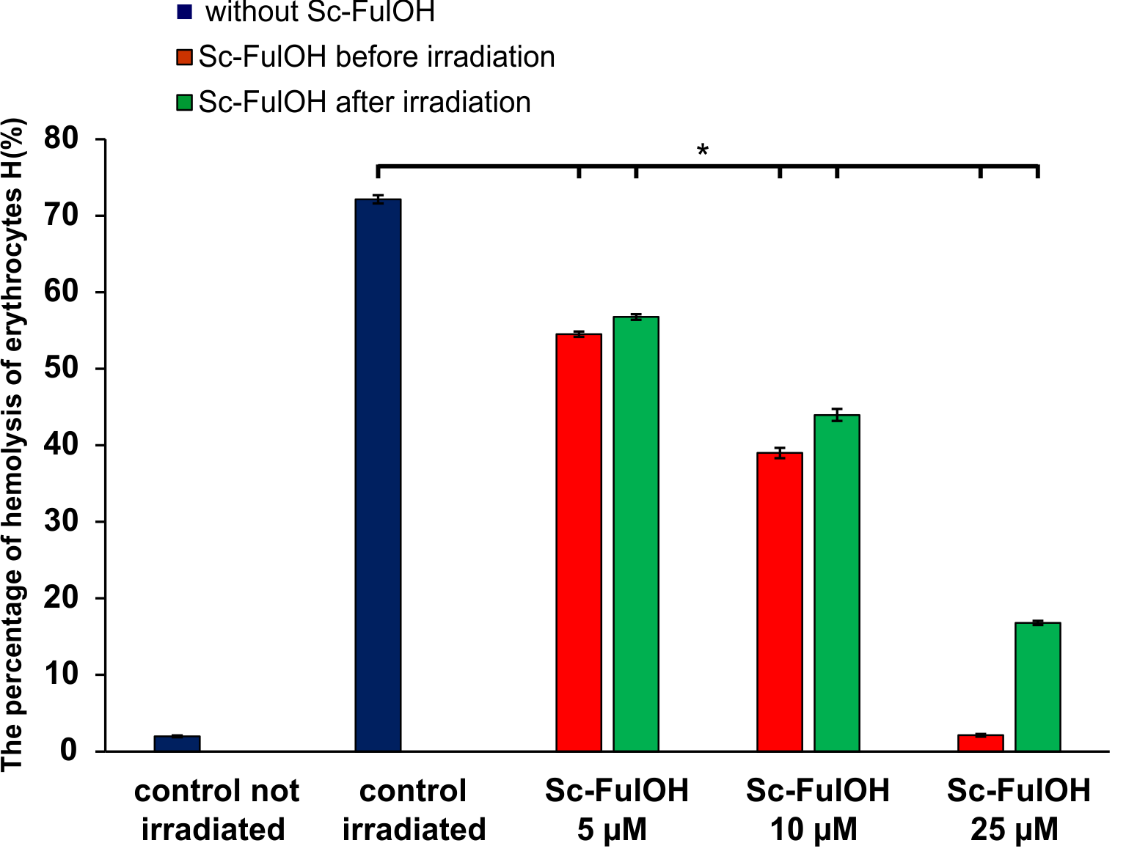


**Figure S1.** Effect of Sc-FulOH on radiation-induced hemolysis of erythrocytes. The percentage
of erythrocyte hemolysis (H%) was measured in the presence of different concentrations of Sc-FulOH
(5, 10, and 25 μM) administered either before or after irradiation. The blue bar represents the control without Sc-FulOH, the red bar represents Sc-FulOH treatment before irradiation, and the green bar represents treatment after irradiation. The non-irradiated control shows minimal hemolysis (~2%), whereas the irradiated control without Sc-FulOH exhibits extensive hemolysis (~72%). The presence
of Sc-FulOH significantly reduces hemolysis compared to the irradiated control, with the strongest protective effect observed at 25 μM. An asterisk (*) indicates a statistically significant difference (*p* < 0.05) compared to the irradiated control.


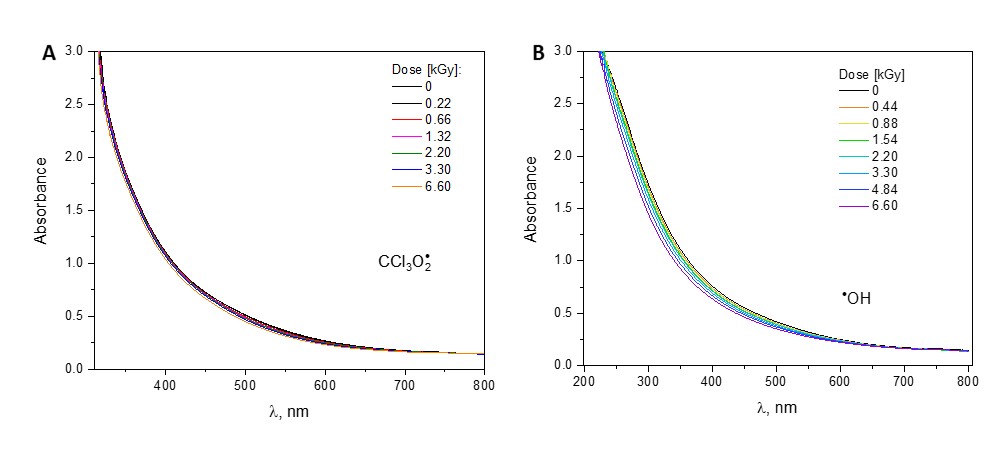


**Figure S2.** UV–Vis spectral analysis of metallofullerenol stability under high-dose irradiation.
Panel **A**: Reaction of metallofullerenol with the CCl_3_OO^•^ radical. Spectra remain unchanged up to 6.6 kGy, demonstrating that the fullerene cage efficiently protects the encapsulated metal from radiation-induced degradation or leakage. Panel **B**: Reaction of metallofullerenol with the hydroxyl radical (HO^•^). Even at 6.6 kGy, no significant changes in the spectra are observed, confirming the robustness of the metallofullerenol structure and the integrity of the fullerene cage under conditions exceeding the maximal dose used in erythrocyte experiments (2.1 kGy).
